# Adalimumab Treatment Modulates Vascular Changes in Hidradenitis Suppurativa Lesions in a Sex-Dependent Manner

**DOI:** 10.3390/biomedicines14040741

**Published:** 2026-03-24

**Authors:** Bepa Pavlić, Marin Ogorevc, Nela Kelam, Ana Stipić, Ema Borovina, Petar Hučić, Ante Čizmić, Dubravka Vuković, Katarina Vukojević, Mirna Saraga-Babić, Snježana Mardešić

**Affiliations:** 1 Department of Dermatology and Venereology, University Hospital of Split, Spinčićeva 1, 21000 Split, Croatia; bpavlic@kbsplit.hr (B.P.); astipic@kbsplit.hr (A.S.); dvukovic@kbsplit.hr (D.V.); 2 Department of Anatomy, Histology and Embryology, University of Split School of Medicine, Šoltanska 2A, 21000 Split, Croatia; nela.kelam@mefst.hr (N.K.); emaborovina5@gmail.com (E.B.); petar.hucic@mefst.hr (P.H.); katarina.vukojevic@mefst.hr (K.V.); msb@mefst.hr (M.S.-B.); smbrakus@gmail.com (S.M.); 3 Department of Pathology, Forensic Medicine, and Cytology, University Hospital Split, 21000 Split, Croatia; acizmic@kbsplit.hr; 4 Center for Translational Research in Biomedicine, University of Split School of Medicine, Šoltanska 2a, 21000 Split, Croatia; 5 Mediterranean Institute for Life Sciences, University of Split, 21000 Split, Croatia

**Keywords:** hidradenitis suppurativa, adalimumab, tumor necrosis factor inhibitors, angiogenesis, CD31 antigen, von Willebrand factor, α-smooth muscle actin, vimentin, Ki-67, cleaved Caspase-3, proliferation, apoptosis, endothelial cells

## Abstract

**Background/Objectives**: Hidradenitis suppurativa (HS) is a chronic, immune-mediated inflammatory skin disease characterized by painful nodules, abscesses, sinus tracts, and progressive fibrosis. Vascular activation is becoming increasingly acknowledged as an important factor in HS pathogenesis; however, the effects of tumor necrosis factor alpha (TNF-α) blockade on vascular remodeling in HS remain poorly characterized. This study investigated the impact of TNF-α inhibition by adalimumab (ADA) on endothelial and fibroblast-associated markers in HS lesions. **Methods**: Formalin-fixed paraffin-embedded skin samples from 71 HS patients were analyzed, including treatment-naive (*n* = 38) and adalimumab-treated (*n* = 33) cases. Histopathology and immunofluorescence were performed using antibodies against CD31, von Willebrand factor (vWF), α-smooth muscle actin (αSMA), vimentin, Ki-67 (proliferation), and cleaved Caspase-3 (apoptosis). ImageJ software was used to determine the immunoexpression of selected markers and vascular density. Vascular density, assessed as vessel count per mm^2^, was designated as the primary endpoint. Sex-related differences were analyzed as exploratory endpoints. **Results**: Adalimumab-treated tissue exhibited significantly reduced vascular density (*p* < 0.01) compared to the treatment-naive group. Conversely, vimentin immunoexpression was significantly higher (*p* < 0.01) in the adalimumab-treated group. No significant differences were found in endothelial Ki-67 or cleaved Caspase-3 expression between treatment groups, indicating that the observed reduction in vascular density is not associated with direct effects on endothelial cell proliferation or apoptosis, but rather may occur indirectly through attenuation of the pro-angiogenic inflammatory milieu. Exploratory sex-stratified analysis revealed that treatment-naive males had significantly higher endothelial proliferation (Ki-67; *p* = 0.031) and vimentin expression (*p* = 0.017) compared to treatment-naive females. In the ADA-treated group, males exhibited significantly lower vascular density (*p* = 0.036) and higher endothelial apoptosis (*p* = 0.039) compared to females, whereas females showed a significant increase in vimentin expression following treatment (*p* = 0.008), suggesting possible sex-dependent differences in vascular remodeling. **Conclusions**: TNF-α blockade is associated with reduced vascular density, consistent with indirect anti-angiogenic effects, suggesting that adalimumab exerts disease-modifying effects on the microenvironment beyond inflammatory cytokine suppression. Sex-dependent differences in vascular regression underscore the importance of considering sex as a biological variable in HS pathogenesis and treatment response. These results highlight the significance of vascular interactions in HS and support adalimumab as a disease-modifying treatment. These exploratory findings require confirmation in longitudinal studies with paired biopsies.

## 1. Introduction

Hidradenitis suppurativa (HS) is a chronic, relapsing, immune-mediated follicular disorder that can severely impact a patient’s quality of life due to pain, scarring, and psychological distress. It predominantly affects intertriginous sites such as the axillae, the inguinogenital and perineal regions, and is characterized by the development of deep nodules, abscesses, draining tunnels, and cicatricial lesions. Follicular occlusion and rupture, with subsequent release of keratinous material, initiate sterile inflammation that is often complicated by secondary infection and progressive fibrotic remodeling [1]. The current consensus among experts regarding HS pathogenesis supports an integrative model in which innate immune networks, consisting of neutrophils, macrophages, as well as interleukin (IL) and tumor necrosis factor (TNF) signaling axes, interact with cutaneous components (keratinocytes, fibroblasts, endothelium) to form self-sustaining “tissue ecosystems” [2]. Single-cell analyses of HS lesions have revealed broad activation of inflammatory pathways (IL-1β, IL-6, IL-8, IL-17, IL-23, TNF-α), stromal signaling (e.g., Hippo pathway), and extracellular matrix (ECM) remodeling processes that correlate with fibrosis [3]. These immune–stromal networks organize tertiary lymphoid structures in chronic HS skin, which depend, in part, on specific TNF-α responsive fibroblast microenvironments that shape local B-cell maturation [4,5]. Within this ecosystem, angiogenesis may act both as a driver and a consequence of chronic disease activity.

Despite growing evidence for vascular involvement in HS pathogenesis, the effects of TNF-α blockade on vascular remodeling in HS lesions remain poorly characterized. In inflamed skin, pro-angiogenic mediators, including vascular endothelial growth factor A (VEGF-A), TNF-α, IL-8, and IL-17, promote endothelial proliferation, differentiation into tip and stalk cells, increased permeability, and perivascular maturation. IL-17, through VEGF upregulation and induction of ECM remodeling, directly promotes angiogenesis and potentiates TNF-α, one of the key clinical and pathogenetic axes in HS [6]. Neutrophil-dominated inflammation further amplifies endothelial activation and microthrombosis, stimulating the release of von Willebrand factor (vWF) from Weibel–Palade bodies and thereby enhancing leukocyte recruitment [7,8,9]. In parallel, complement activation also interacts with TNF-α signaling, contributing to vascular-inflammatory crosstalk [10]. Therapeutically, TNF-α blockade with adalimumab (ADA) induces anti-angiogenic or “vascular-normalizing” effects in chronic dermatoses. In psoriasis, ADA reduces endothelial proliferation, vascular density, and vessel caliber in parallel with clinical improvement, suggesting that biologic agents, such as TNF inhibitors, can play a role in remodeling the tissue microvasculature [11]. Beyond psoriasis, anti-TNF agents can modify vascularization in a range of systemic inflammatory diseases, supporting the hypothesis that similar effects may influence vascular architecture and permeability in HS lesions [12]. Given the growing evidence for IL-17’s involvement in key etiopathogenetic mechanisms of HS, it is most likely that the interplay between TNF and IL-17 affects the vasculature in characteristic HS lesions such as tunnels and plaques [6].

To investigate vascular and stromal changes in HS, we selected a panel of four complementary markers, each addressing a specific and non-redundant aspect of vascular or stromal biology. Cluster of differentiation 31 (CD31), also known as platelet endothelial cell adhesion molecule-1 (PECAM-1), is a junctional adhesion molecule expressed on the surface of endothelial cells and some leukocytes, where it plays an essential role in leukocyte diapedesis and vascular integrity. In histological studies, CD31 is widely used as a reliable marker for identifying microvessels and evaluating their morphometric features, including density, area, and diameter [13]. vWF, stored in endothelial Weibel–Palade bodies, is a key endothelial glycoprotein whose regulated release and increased expression in HS lesions reflect endothelial activation and microthrombosis, linking chronic inflammation to vascular remodeling [14,15,16]. In the pathogenesis of HS, α-smooth muscle actin (αSMA) plays a dual role and can be used as a marker of both angiogenesis and fibrosis. It marks pericytes and vascular smooth muscle cells (VSMCs) as contractile components of the vessel wall and is essential for evaluating pericyte coverage and maturation, as well as vascular stability. αSMA is also expressed by myofibroblasts, and the number of these contractile fibroblasts increases during the formation of tunnels and other fibrotic lesions [17,18,19]. Vimentin is an intermediate filament protein widely expressed in mesenchymal cells, including fibroblasts and myofibroblasts, where it regulates migration, extracellular matrix organization, and tissue remodeling. In HS, increased vimentin expression has been associated with mesenchymal cell activity and fibrotic processes [4]. Importantly, vimentin was included in our panel as a marker of general mesenchymal and stromal activity rather than as a specific endothelial marker. While colocalization of vimentin with the endothelial marker CD31 may suggest endothelial–mesenchymal transition (EndMT), this interpretation requires confirmation with additional markers and functional assays.

Ki-67 is a large, cell cycle–regulated nuclear protein expressed during all active phases of the cell cycle (G1, S, G2, and M) and is widely used in histopathology as a universal marker of cellular proliferation [20]. While Ki-67 has predominantly been studied in keratinocytes, its potential role in endothelial cell dynamics, especially in inflammatory dermatoses such as HS has also been increasingly discussed [20,21]. Caspases are key regulators of programmed cell death, and cleaved Caspase-3, generated by proteolytic cleavage at Asp175, is commonly used to identify cells that have committed to apoptosis [22,23]. In HS, cleaved Caspase-3 expression has not been widely investigated. By including Ki-67 and cleaved Caspase-3 in our analysis, we aimed to determine whether any observed changes in vascular density are associated with direct effects on endothelial cell proliferation or apoptosis, or whether they reflect indirect effects through modification of the inflammatory microenvironment.

Considering the central role of angiogenesis and vascular remodeling in sustaining chronic cutaneous inflammation, the established role of TNF-α in HS pathogenesis, and clinical evidence that anti-TNF therapy can normalize the microvasculature, we hypothesized that vascularization would be decreased in HS lesions of ADA-treated patients, compared to biologic-naive patients. Therefore, the aim of this study was to determine protein expression by immunofluorescent staining and image analysis of CD31, vWF, αSMA, and vimentin in HS lesions from biologic treatment-naive and ADA-treated patients, and to assess endothelial proliferation and apoptosis as potential mechanisms underlying vascular changes. Our findings may provide further insight into the role of the vascular microenvironment in the pathogenesis of HS and enhance understanding of the vascular effects of ADA treatment.

## 2. Materials and Methods

### 2.1. Tissue Procurement and Processing

All study procedures were approved by the Ethical and Drug Committee of the University Hospital of Split (class: 500-03/23-01/238; registry number: 2181-147/01/06/LJ.Z.-23-02) and were conducted in accordance with the Declaration of Helsinki and all relevant institutional and national guidelines and regulations.

Skin biopsy specimens were obtained from patients diagnosed with hidradenitis suppurativa at the Department of Dermatovenereology, University Hospital of Split, after written informed consent was obtained from each participant. The diagnosis of hidradenitis suppurativa was confirmed both clinically and histopathologically, in accordance with established diagnostic criteria. Samples were collected between January 2023 and December 2024 from a total of 71 patients and were divided into two groups: ADA-treated (*n* = 33) and treatment-naive (*n* = 38). Patients with other inflammatory diseases, systemic autoimmune conditions, or ongoing antibiotic or corticosteroid therapy were excluded from the study. Eligibility criteria included duration of ADA treatment for at least 12 weeks for participants in the ADA-treated group, the availability of sufficient formalin-fixed paraffin-embedded (FFPE) material for immunohistochemical (IHC) analysis, and complete accompanying clinical documentation. Cases with incomplete laboratory data or inadequate tissue material for IHC examination were excluded from the study. All patients in the ADA-treated group received the standard approved dosing regimen for HS, consisting of 160 mg at week 0, 80 mg at week 2, followed by 80 mg every two weeks, in accordance with the approved label and institutional protocol. ADA trough levels and anti-drug antibody measurements were not available for this cohort. Control (CTRL) skin samples shown in histopathological comparisons were obtained from non-involved perilesional skin margins during surgical excision procedures in HS patients, representing non-inflamed reference tissue.

Tissue specimens were systematically processed in the pathology laboratory applying conventional histological techniques, which involved fixation in 4% paraformaldehyde (PFA) formulated in 0.1 M phosphate-buffered saline (PBS), dehydration via a graded ethanol series, paraffin embedding, and sectioning using a rotary microtome (RM2125 RTS, Leica, Buffalo Grove, IL, USA). Sections were affixed to glass slides for hematoxylin-eosin (H&E) staining, histochemical collagen staining, and immunofluorescence examination. To qualitatively assess collagen deposition, representative sections from control, treatment-naive, and ADA-treated groups were stained with Picrosirius Red and Mallory’s Trichrome using standard protocols. Picrosirius Red staining highlights collagen fibers in red against a pale yellow background, while Mallory’s Trichrome differentiates collagen (blue) from muscle fibers and cytoplasm (red) and nuclei (dark brown/black). Stained sections were examined under a light microscope (BX40, Olympus, Tokyo, Japan) for qualitative assessment of fibrotic changes.

### 2.2. Immunofluorescence Staining

The immunofluorescence procedure was carried out as previously described [24,25]. Briefly, after deparaffinization in xylene and rehydration through a graded ethanol series, tissue sections mounted on glass slides underwent heat-induced antigen retrieval by incubation in 0.01 M citrate buffer (pH 6.2) at 95 °C for 30 min using a water steamer, followed by cooling to room temperature. The sections were subsequently washed with 0.1 M PBS and treated with a commercial protein blocking reagent (ab64226, Abcam, Cambridge, UK) for 20 min to reduce nonspecific antibody binding.

Primary antibodies were administered to the sections, which were then incubated overnight in a humidity-regulated chamber (StainTray slide staining system; Sigma-Aldrich, St. Louis, MO, USA) (Table 1). The subsequent day, slides were rinsed with PBS and treated with suitable secondary antibodies for one and a half hours at ambient temperature (Table 1). Nuclear staining was conducted utilizing 4′,6-diamidino-2-phenylindole (DAPI) subsequent to a final rinse with PBS. Ultimately, sections were air-dried and affixed with Immuno-Mount media (Thermo Shandon, Pittsburgh, PA, USA) to safeguard the materials for microscopic examination.

Isotype-matched controls and secondary antibody-only samples were employed to mitigate nonspecific background signals. Isotype-matched controls involved substituting the primary antibody with a non-target-specific antibody of identical isotype, thereby enabling the assessment of potential nonspecific binding. In controls with only secondary antibodies, the primary antibody was omitted to identify any nonspecific binding of the secondary antibody. These controls ensure that the detected signals were specific to the target protein and uninfluenced by background noise, including residual paraffin.

### 2.3. Imaging and Data Processing of Histological and Immunofluorescence Sections

Histological assessment of hematoxylin and eosin (H&E) stained sections was conducted utilising a light microscope (BX40, Olympus, Tokyo, Japan). Immunofluorescence imaging was performed using a BX51 Olympus microscope, integrated with a Nikon DS-Ri2 digital camera (Nikon Corporation, Tokyo, Japan) and NIS-Elements F software (version 3.0). To evaluate the immunoexpression of CD31 and vimentin in the examined samples, 10 non-overlapping representative fields per section were photographed at 40× magnification, maintaining uniform exposure settings throughout all samples. Quantitative image analysis was conducted utilising ImageJ software (version 1.51; NIH, Bethesda, MD, USA). Area percentage measures of the positive signal were derived by median filter subtraction and colour thresholding, as previously outlined [26,27,28,29]. The “area percentage” denotes the ratio of the image occupied by the positive fluorescent signal, determined by the count of fluorescent pixels exceeding a specified threshold divided by the total pixel count in the image. Importantly, the quantification was conducted across the whole visual field and did not differentiate between specific cell types (e.g., endothelial vs. stromal cells); rather, the positive signal was assessed across designated regions of interest. As such, the vimentin area percentage reflects total mesenchymal signal in the analyzed field, encompassing both endothelial and stromal compartments. The results were averaged for each assessed sample.

For samples stained with vWF and αSMA to evaluate vascularization, the average number of blood vessel sections per mm^2^ was assessed as follows. Whole-slide images of stained sections were acquired using an Olympus BX51 microscope equipped with a Nikon DS-Ri2 camera, using NIS-Elements F software and Microvisioneer Manual WSI 2021C-32 (Microvisioneer GmbH, Ledererzeile, Germany). The total tissue surface area for each sample was determined by manually outlining the region of interest (ROI) using the Polyline tool in the QuPath open-source digital pathology software (version 0.5.1) [30]. Images were then exported as high-resolution TIFF files and analysed in ImageJ (National Institutes of Health, Bethesda, MD, USA). Blood vessel sections were identified and manually counted using the Cell Counter plugin. Blood vessel sections were distinguished from sweat glands and other structures based on specific vWF immunoreactivity localized to endothelial cells, which clearly delineated vascular profiles. The total number of identified blood vessel sections was divided by the corresponding tissue area (in mm^2^) to calculate the average vessel density (vessels/mm^2^). Vessel counting was performed by one investigator and independently validated by a second observer on a random subset of samples to assess inter-observer agreement. All quantitative data were tabulated in Microsoft Excel (Microsoft Corp., Redmond, WA, USA) prior to statistical analysis.

### 2.4. Assessment of Endothelial Cell Proliferation and Apoptosis

To investigate the mechanisms underlying the observed reduction in vascular density in ADA-treated lesions, additional immunofluorescence analyses were performed using antibodies against Ki-67 and cleaved Caspase-3 (Asp175). Both markers were evaluated exclusively in endothelial cells of blood vessels within HS lesions.

The immunofluorescence staining protocol was performed as described in Section 2.2, using double staining with Ki-67 or cleaved Caspase-3 in combination with the endothelial marker CD31. For quantification, blood vessels were identified in immunofluorescence sections based on characteristic vascular morphology and CD31 immunoreactivity. For each sample, approximately 7 blood vessel cross-sections (range: 5–12) were analyzed. The number of evaluable vessel profiles was determined by the availability of well-oriented vessel cross-sections within each tissue section, rather than by a predetermined sampling target. Within each vessel profile, individual endothelial cells were classified as positive (exhibiting nuclear Ki-67 immunoreactivity or cytoplasmic cleaved Caspase-3 immunoreactivity) or negative based on the colocalization with CD31. The proliferation index was defined as the percentage of Ki-67-positive endothelial cells per vessel, and the apoptosis index as the percentage of cleaved Caspase-3-positive endothelial cells per vessel. For each patient, the mean percentage across all analyzed vessels was calculated and used as the per-patient value for statistical analysis.

### 2.5. Statistical Analysis

Statistical analysis was performed using GraphPad Prism (version 10.5.0; GraphPad Software, San Diego, CA, USA). Results were considered statistically significant at *p* < 0.05. Data are presented as mean ± standard deviation (SD). Graphical representations were created in GraphPad Prism, and illustrative plates were assembled using Adobe Photoshop v9.0 (CS2) (Adobe Systems, San Jose, CA, USA).

Differences in age between the two groups were evaluated using the unpaired *t*-test with Welch’s correction. Differences between groups according to sex and biopsy site (axilla vs. other sites—pubic, inguinal, gluteal) were analyzed using contingency table analysis (Chi-square test with Fisher’s exact correction for small sample sizes).

Quantitative differences between treatment-naive and ADA-treated groups were analyzed using the Mann–Whitney U test for all markers. Each data point represents one biopsy per patient, with values averaged from multiple microscopic fields or vessel profiles. Vascular density (vessels/mm^2^, assessed by vWF/αSMA staining) was designated as the primary endpoint. Vimentin area percentage, Ki-67 proliferation index, and cleaved Caspase-3 apoptosis index were designated as secondary endpoints. All sex-stratified analyses were designated as exploratory. To explore potential sex-related differences in vascular remodeling, quantitative assessments were conducted separately for male and female patients in both treatment-naive and ADA-treated groups using the Mann–Whitney U test. Each data point represents one biopsy per patient, with values averaged from multiple microscopic fields or vessel profiles. Given the exploratory nature of the sex-stratified analyses and the modest subgroup sizes, Benjamini–Hochberg false discovery rate (FDR) correction was applied across all sex-stratified comparisons (m = 10). Given that none of the originally significant *p*-values survived FDR correction, these findings should be interpreted with caution and require replication in larger cohorts.

A post hoc power analysis was performed using GraphPad Prism. For the primary comparison of vascular density between treatment-naive (*n* = 38) and ADA-treated (*n* = 33) groups, with α = 0.05 and power = 0.80, the minimum detectable effect size was Cohen’s d = 0.67, indicating that the study was adequately powered to detect medium-to-large between-group differences in the primary endpoint (observed Cohen’s d = 0.70).

## 3. Results

### 3.1. Clinical Data and Histopathological Analysis of Control and Hidradenitis Suppurativa Skin

The main clinical data of the patient cohort are described in Table 2. There were no statistically significant differences between the two groups regarding sex (*p* = 1.000), age (*p* = 0.100), BMI (*p* = 0.312), smoking status (*p* = 0.409), or biopsy region (axilla vs. other sites—pubic, inguinal, gluteal; *p* = 0.510). However, the ADA-treated group had significantly higher Hurley stage distribution (*p* = 0.007), higher IHS4 scores (12.2 ± 7.2 vs. 8.5 ± 7.4; *p* = 0.021), and longer disease duration (12.2 ± 10.0 vs. 6.6 ± 7.0 years; *p* = 0.002) compared to the treatment-naive group. These differences are expected, as adalimumab is prescribed for patients with moderate-to-severe disease who have not responded to conventional therapy. The mean duration of adalimumab treatment was 2.1 ± 1.1 years (range: 0.5–4.0 years).

**Figure 1:**
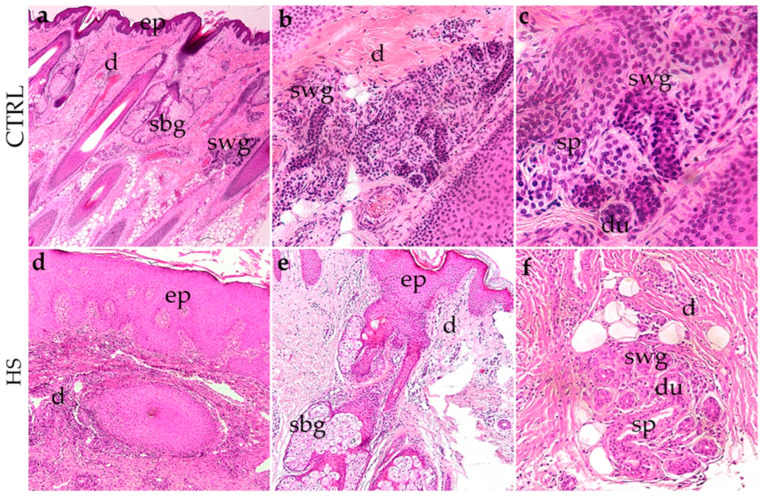
Comparative histopathology of control skin (CTRL; (**a**–**c**)) versus hidradenitis suppurativa (**d**–**f**) stained using hematoxylin and eosin (H&E). (**a**–**c**) Normal skin displays a well-organized epidermis (ep), dermis (**d**), sebaceous glands (sbg), and sweat glands (swg) with undamaged ducts (du). Secretory portions (sp) of sweat glands are clearly visible in the dermal structure. (**d**–**f**) Hidradenitis suppurativa lesions reveal hyperplasia of the epidermis and pronounced inflammatory infiltration within the dermis (**d**,**e**). Sweat glands (swg) and duct (du) structures are distorted, showing disrupted secretory portions (sp) and surrounding fibrosis. Infiltration of adipose tissue and dermal thickening are evident, consistent with the chronic inflammatory changes and glandular damage observed in hidradenitis suppurativa. The most characteristic elements noticed for each phenotype are shown in inserts corresponding to the dashed boxes. Images were taken at a magnification of ×4 (**a**), ×10 (**d**,**e**), ×20 (**b**,**f**), and ×40 (**c**).

The histological assessment of control (CTRL) skin samples, obtained from non-involved perilesional skin margins during surgical excision, revealed a typical epidermal structure characterized by a consistently layered squamous epithelium with a preserved basal layer and well-organized keratinocyte maturation (Figure 1a). No signs of inflammatory cell infiltration or disorganized tissue architecture were noted (Figure 1a). The dermis presented a regular connective tissue composition with well-defined sebaceous and sweat glands, which included intact ducts and secretory portions (Figure 1b,c).

In stark contrast, skin samples from patients with hidradenitis suppurativa exhibited significant pathological changes. The epidermis showed irregular thickening (acanthosis) and hyperplasia, with localized areas of parakeratosis and thickening of the stratum spinosum (Figure 1d). The dermis exhibited extensive inflammatory cell infiltration, mainly comprised of lymphocytes and neutrophils, surrounding and infiltrating around the dermal appendages (Figure 1d). The sweat glands and their ducts were notably distorted and frequently enveloped by dense inflammatory infiltrates and fibrotic tissue (Figure 1e,f). The secretory portions appeared disrupted or degenerated, with glandular remnants embedded in fibrotic stroma interspersed with adipose tissue in some regions (Figure 1e,f). These histopathological findings indicate a state of chronic inflammation, glandular damage, and ongoing tissue remodeling that is characteristic of advanced hidradenitis suppurativa lesions.

Picrosirius Red and Mallory’s Trichrome staining were performed on representative samples to qualitatively assess collagen deposition across groups (Supplementary Figures S1 and S2). In control skin, both stains revealed organized collagen fibers surrounding adnexal structures, with a balanced ratio of collagen to cellular components. Treatment-naive HS lesions exhibited markedly increased collagen deposition, with dense staining throughout the dermis indicating extensive fibrosis and compression of glandular structures. In ADA-treated samples, collagen deposition appeared intermediate between control and treatment-naive tissue, with less dense staining and better-preserved glandular architecture compared to treatment-naive lesions. These qualitative observations complement the immunofluorescence findings and are consistent with reduced fibrotic remodeling in ADA-treated HS skin.

### 3.2. Vascular Density in Hidradenitis Suppurativa Skin Lesions

**Figure 2:**
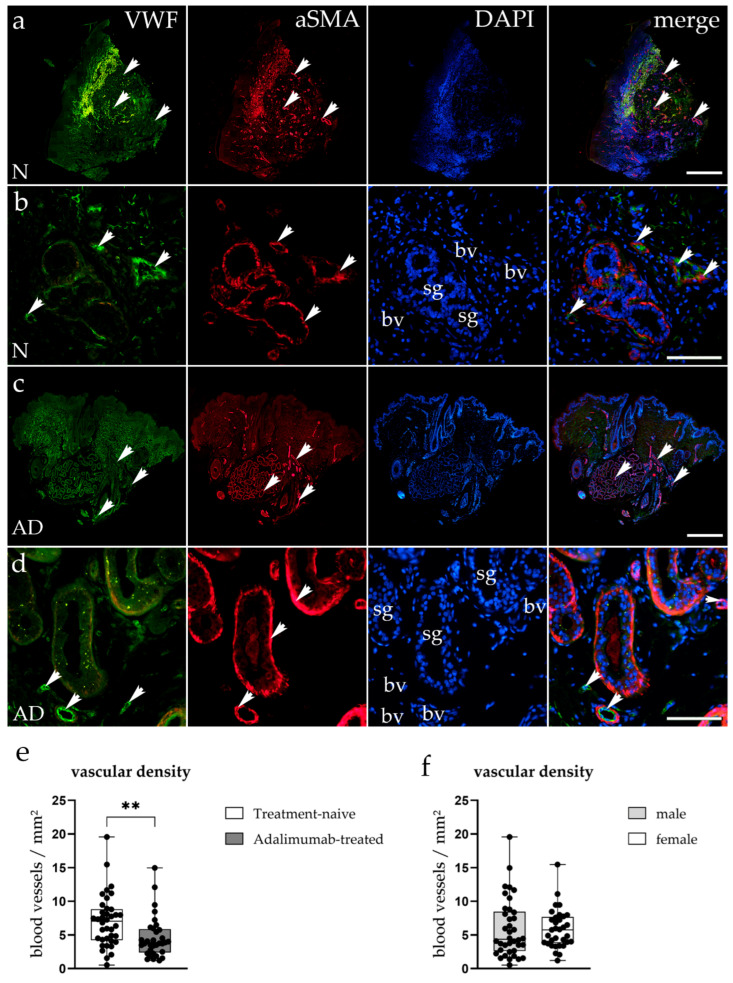
Immunofluorescent staining of vascular markers in treatment-naive and ADA-treated skin. (**a**–**d**) Representative images showing von Willebrand factor (vWF, green), α-smooth muscle actin (αSMA, red), and nuclei (DAPI, blue) in treatment-naive (N; (**a**,**b**)) and ADA-treated (AD; (**c**,**d**) HS lesions. Merged images demonstrate colocalization of endothelial (vWF^+^) and perivascular smooth muscle (αSMA^+^) cells forming blood vessel (bv) walls (arrowheads), while sweat glands (sg) are αSMA^+^ and VWF^−^. Treatment-naive samples show dense and irregular vasculature, while ADA-treated tissue exhibits reduced vascular density and weaker vWF/αSMA signals. Images (**a**,**c**) show the entire sample (scale bar: 500 μm), while images (**b**,**d**) were captured at 40× magnification (scale bar: 100 μm). (**e**,**f**) Quantification of blood vessel density (vessels/mm^2^) reveals a significant reduction in the ADA-treated group compared to treatment-naive lesions (** *p* < 0.01). There was no significant difference in blood vessel density between sexes.

Immunofluorescence staining targeting vWF and αSMA was used to visualize endothelial cells and perivascular smooth muscle cells in hidradenitis suppurativa lesions from treatment-naive and ADA-treated patients to determine vascular density (Figure 2a–d). In the treatment-naive HS tissue, vWF-positive blood vessels were plentiful throughout the dermis, frequently surrounding sweat glands (Figure 2a,b). These vessels demonstrated strong αSMA expression, indicating substantial vascular remodeling and perivascular activation in inflamed zones (Figure 2a,b). Merged images illustrated a significant spatial overlap of vWF and αSMA signals, reflecting hypervascularization and endothelial activation typical of chronic inflammatory responses (Figure 2a,b). Conversely, ADA-treated HS samples exhibited a noticeable reduction in both vWF and αSMA staining intensity, along with fewer vascular structures overall (Figure 2c,d). The remaining vessels appeared less dilated and more uniformly distributed, findings consistent with partial reduction in vascular density following TNF-α inhibition (Figure 2c,d). Quantitative assessment confirmed a significant reduction in average vascular density (vessels/mm^2^) in ADA-treated samples compared to treatment-naive lesions (*p* < 0.01) (Figure 2e).

### 3.3. Proliferation of Endothelial Cells in Hidradenitis Suppurativa Skin Lesions

**Figure 3:**
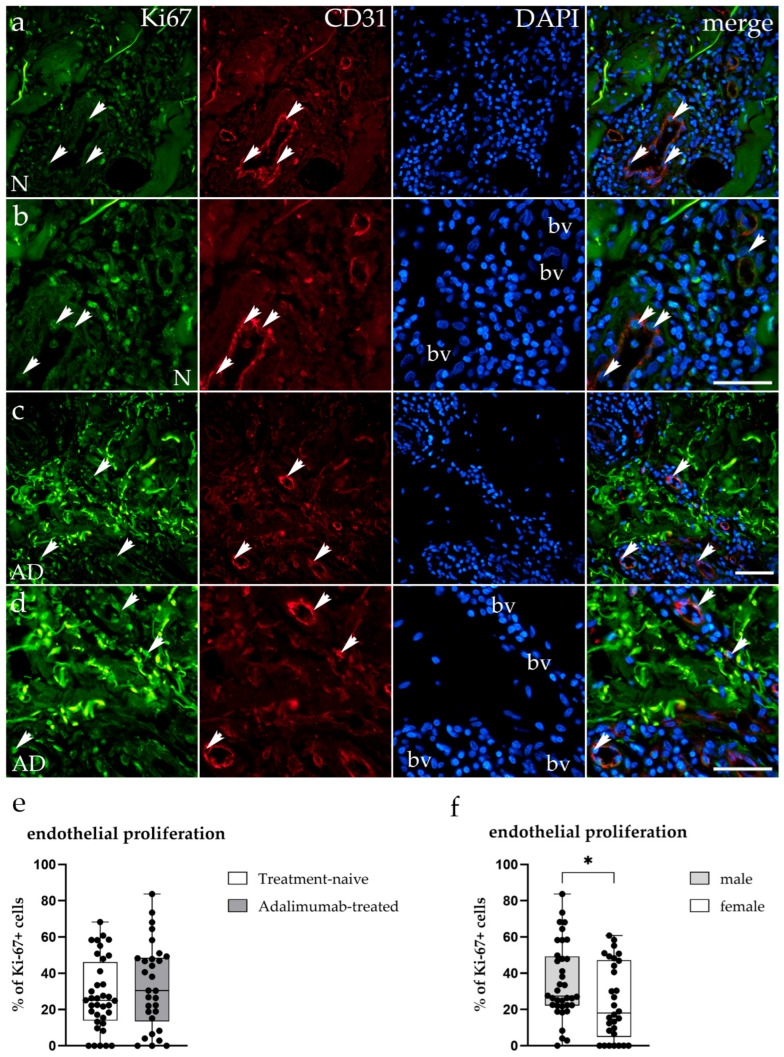
Immunofluorescent staining of the proliferation marker Ki-67 in treatment-naive and ADA-treated hidradenitis suppurativa skin. (**a**–**d**) Representative immunofluorescent images showing Ki-67 (green), platelet endothelial cell adhesion molecule-1 (PECAM-1, CD31) (red), and nuclei (DAPI–4′,6-diamidino-2-phenylindole, blue) in treatment-naive (N; (**a**,**b**)) and ADA-treated (AD; (**c**,**d**)) HS lesions. Arrowheads mark Ki-67^+^ endothelial cells within blood vessel (bv) walls displaying nuclear Ki-67 immunoreactivity co-localizing with the endothelial marker CD31. Treatment-naive samples show proliferating endothelial cells in abundant vascular structures, while ADA-treated tissue exhibits fewer vascular structures with comparable endothelial Ki-67 expression. Images (**a**,**c**) were captured at 20× magnification and images (**b**,**d**) at 40× magnification. A scale bar of 100 μm applies to all images. (**e**) Quantification of the percentage of Ki-67-positive endothelial cells reveals no significant difference between treatment-naive and ADA-treated groups (*p* = 0.44). (**f**) Sex-stratified analysis within the treatment-naive group shows significantly higher endothelial Ki-67 expression in males compared to females (*p* = 0.031). Data are presented as mean ± SD. Statistical significance was determined using the Mann–Whitney U test. * *p* < 0.05.

To determine whether the observed reduction in vascular density in ADA-treated HS lesions was associated with changes in endothelial cell proliferation, double immunofluorescence staining for Ki-67 (green) and CD31 (red) was performed on tissue samples from treatment-naive and ADA-treated patients (Figure 3a–d).

In treatment-naive HS tissue, Ki-67-positive endothelial cells were observed within blood vessel walls, indicating ongoing proliferative activity in the dermal microvasculature (Figure 3a,b). CD31-positive vascular structures were abundant, with Ki-67^+^ endothelial cells detected in both small- and large-caliber vessels (Figure 3b). In ADA-treated samples, Ki-67-positive endothelial cells were similarly present within the remaining blood vessels, although vascular structures were fewer overall (Figure 3c,d). Merged images confirmed the colocalization of Ki-67 and CD31 signals within the endothelium of blood vessels in both groups (Figure 3a–d). Quantitative analysis revealed no significant difference in the percentage of Ki-67-positive endothelial cells between ADA-treated (33.13 ± 23.55%) and treatment-naive (27.95 ± 19.69%) groups (*p* = 0.44; Figure 3e), indicating that the reduction in vascular density is not associated with suppression of endothelial cell proliferation. However, within the treatment-naive group, males exhibited a significantly higher Ki-67 proliferation index than females (33.04 ± 15.84% vs. 21.59 ± 22.55%; *p* = 0.031; Figure 3f), suggesting sex-dependent differences in endothelial proliferative activity.

### 3.4. Apoptosis of Endothelial Cells in Hidradenitis Suppurativa Skin Lesions

**Figure 4:**
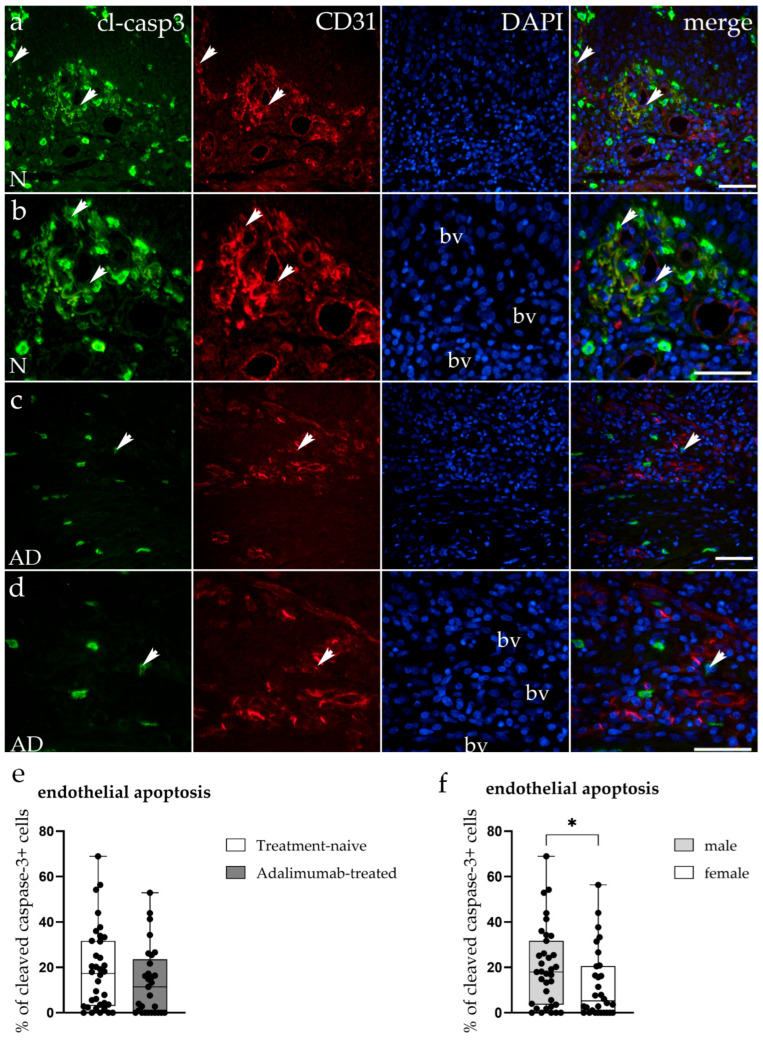
Immunofluorescent staining of the apoptosis marker cleaved Caspase-3 in treatment-naive and ADA-treated hidradenitis suppurativa skin. (**a**–**d**) Representative immunofluorescent images showing cleaved Caspase-3 (Asp175) (green), platelet endothelial cell adhesion molecule-1 (PECAM-1, CD31) (red), and nuclei (DAPI–4′,6-diamidino-2-phenylindole, blue) in treatment-naive (N; (**a**,**b**)) and ADA-treated (AD; (**c**,**d**)) HS lesions. Arrowheads mark cleaved Caspase-3^+^ endothelial cells within blood vessel (bv) walls displaying cytoplasmic cleaved Caspase-3 immunoreactivity co-localizing with the endothelial marker CD31. Treatment-naive samples show apoptotic endothelial cells within abundant vascular structures, while ADA-treated tissue exhibits fewer vascular structures with comparable endothelial cleaved Caspase-3 expression. Images (**a**,**c**) were captured at 20× magnification and images (**b**,**d**) at 40× magnification. A scale bar of 100 μm applies to all images. (**e**) Quantification of the percentage of cleaved Caspase-3-positive endothelial cells reveals no significant difference between treatment-naive and ADA-treated groups (*p* = 0.16). (**f**) Sex-stratified analysis within the ADA-treated group shows significantly higher endothelial cleaved Caspase-3 expression in males compared to females (*p* = 0.039). Data are presented as mean ± SD. Statistical significance was determined using the Mann–Whitney U test. * *p* < 0.05.

To assess whether increased endothelial apoptosis contributes to the reduced vascular density in ADA-treated HS lesions, double immunofluorescence staining for cleaved Caspase-3 (green) and CD31 (red) was performed on tissue samples from treatment-naive and ADA-treated patients (Figure 4a–d). Cleaved Caspase-3 is the activated form of the executioner caspase generated by proteolytic cleavage at Asp175 and specifically detects cells that have irreversibly committed to apoptotic cell death. In treatment-naive HS tissue, cleaved Caspase-3-positive cells were observed both within blood vessel endothelium and in the surrounding stroma (Figure 4a,b). CD31-positive vascular structures were abundant, and merged images confirmed colocalization of cleaved Caspase-3 and CD31 signals in endothelial cells undergoing apoptosis (Figure 4a,b). In ADA-treated samples, cleaved Caspase-3 expression was similarly detected in endothelial cells of the remaining blood vessels, though overall vascular density was visibly reduced (Figure 4c,d). Quantitative analysis revealed no significant difference in the percentage of cleaved Caspase-3-positive endothelial cells between treatment-naive (18.72 ± 18.07%) and ADA-treated (13.65 ± 15.13%) groups (*p* = 0.16; Figure 4e), indicating that the reduction in vascular density is not driven by increased endothelial apoptosis. When comparing sexes within the ADA-treated group, males exhibited significantly higher cleaved Caspase-3 expression compared to females (19.86 ± 17.35% vs. 6.99 ± 8.78%; *p* = 0.039; Figure 4f), indicating sex-dependent differences in endothelial apoptotic activity following TNF-α blockade.

### 3.5. Expression of the Mesenchymal Marker Vimentin in the Hidradenitis Suppurativa Skin Lesions

**Figure 5:**
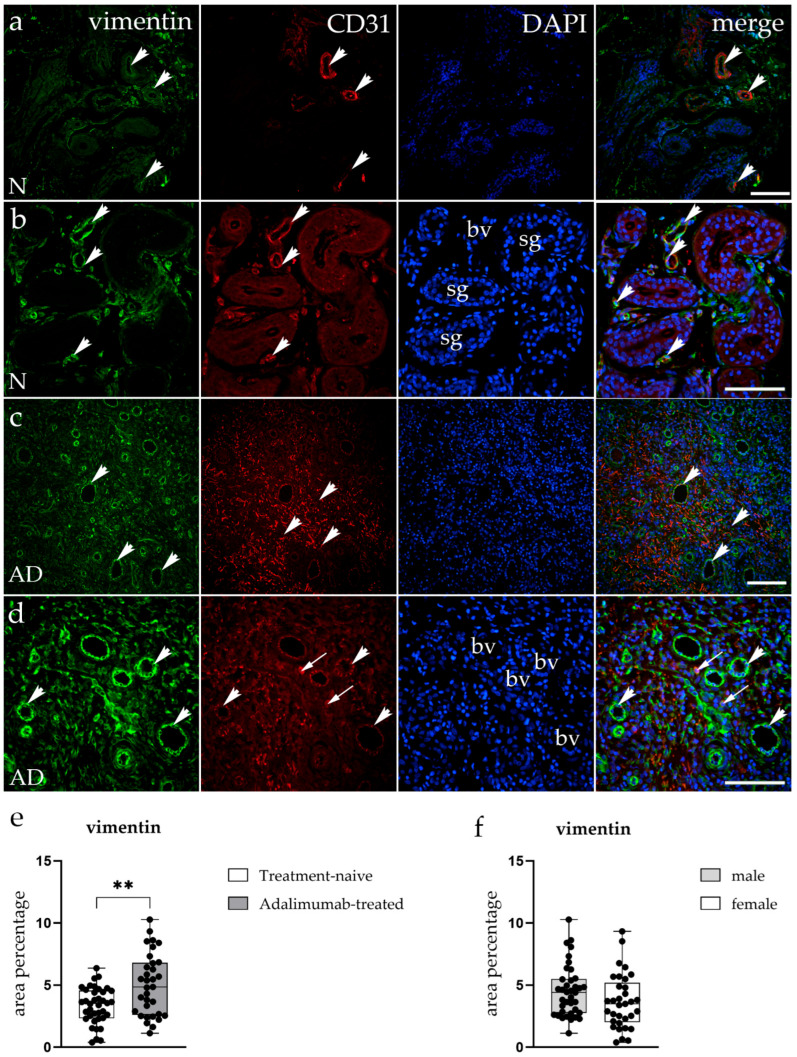
Immunofluorescent staining of the mesenchymal marker vimentin and CD31 in hidradenitis suppurativa skin. (**a**–**d**) Representative immunofluorescent images showing vimentin (green), platelet endothelial cell adhesion molecule-1 (PECAM-1, CD31) (red), and nuclei (DAPI–4′,6-diamidino-2-phenylindole, blue) in treatment-naive (N; (**a**,**b**)) and ADA-treated (AD; (**c**,**d**)) HS lesions. Arrowheads mark areas of colocalization between stromal (vimentin^+^) and endothelial (CD31^+^) cells within the dermis (**d**), while arrows mark CD31+ cells outside of vascular structures. Treatment-naive samples display moderate vimentin expression and numerous CD31^+^ vascular structures surrounding sweat glands (sg) and blood vessels (bv). In ADA-treated tissue, CD31 signal intensity and vessel density are markedly reduced, with diffuse strong vimentin staining. (**e**,**f**) Quantification of vimentin area percentage reveals a significant increase in vimentin expression in ADA-treated lesions (** *p* < 0.01). There was no significant difference in vimentin area percentage between sexes. Images (**a**,**c**) were captured at 20× magnification and images (**b**,**d**) at 40× magnification. A scale bar of 100 μm applies to all images.

To further characterize vascular remodeling in hidradenitis suppurativa, double immunofluorescence staining for vimentin (green) and CD31 (red) was performed on tissue samples from treatment-naive and ADA-treated patients (Figure 5a–d). In treatment-naive lesions, vimentin expression was observed in endothelial and stromal cells throughout the dermis, indicating the presence of fibroblasts and mesenchymal cells within inflamed areas (Figure 5a,b). CD31-positive endothelial cells were abundant and frequently localized around sweat glands, forming irregular and dilated vascular structures. Merged images showed areas of overlap between vimentin and CD31 signals in endothelial cells of vascular structures (Figure 5a,b). In contrast, ADA-treated HS samples showed reduced CD31 staining intensity and fewer vascular structures. Vimentin expression, however, was more extensive, with higher staining intensity observed in both endothelial and stromal cells (Figure 5c,d). Additionally, several cells expressing the endothelial marker CD31 were observed in the stroma, outside vascular spaces (Figure 5d). Quantitative analysis confirmed a significant increase in vimentin-positive area (*p* < 0.01) in the ADA-treated group compared to treatment-naive lesions (Figure 5e). As this quantification was performed across whole visual fields without compartment-specific analysis, the increased vimentin signal encompasses both stromal and endothelial contributions and cannot be attributed to a specific cell type.

### 3.6. Sex-Dependent Differences of Adalimumab-Induced Vascular Changes in Hidradenitis Suppurativa Skin Lesions

**Figure 6:**
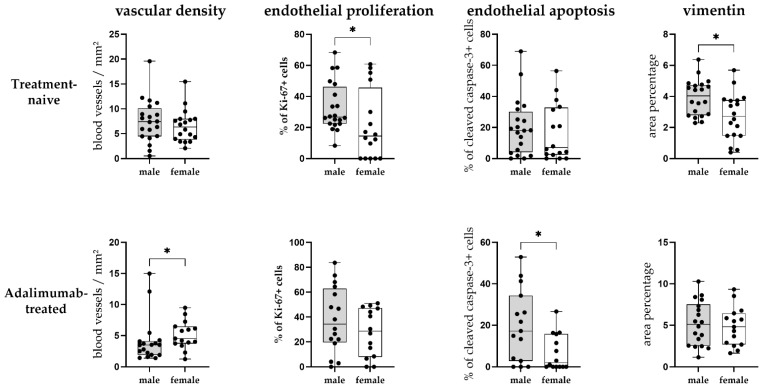
Quantitative analysis of the analysed markers by sex in treatment-naive and ADA-treated hidradenitis suppurativa (HS) lesions. There was no significant difference in vascular density between sexes in treatment-naive HS lesions; however, males had a significantly lower vascular density in ADA-treated HS lesions. Males had significantly higher endothelial proliferation in treatment-naive HS lesions, but no significant difference between sexes was observed in ADA-treated lesions. Regarding endothelial cell apoptosis, no differences were observed between sexes in treatment-naive lesions, whereas males showed significantly higher endothelial apoptosis than females in ADA-treated lesions. Vimentin expression was significantly higher in males of the treatment-naive group, while no significant difference in vimentin expression between sexes was found in the ADA-treated group. Data are presented as mean ± SD. Statistical significance was determined using the Mann–Whitney U test. * *p* < 0.05.

To investigate potential sex-related differences in vascular remodeling, quantitative analysis of vascular density, as well as endothelial proliferation, apoptosis, and vimentin expression, was performed separately for male and female patients within treatment-naive and ADA-treated groups (Figure 6). The following sex-stratified analyses are exploratory and should be interpreted with caution given the modest subgroup sizes (treatment-naive: males *n* = 20, females *n* = 18; ADA-treated: males *n* = 18, females *n* = 15).

While no significant difference in vascular density was initially found between the sexes (Figure 2f), when analysing the treatment-naive and ADA-treated groups separately, we have found that males in the ADA-treated group had a significantly lower vascular density compared to females (*p* = 0.036, BH-adjusted *p* = 0.090; Figure 6), indicating that the decreased vascular density of the ADA-treated group (Figure 2e) is mostly attributable to male samples.

Regarding endothelial proliferation, while no significant difference was found between the treatment groups overall (Figure 3e), sex-stratified analysis revealed that Treatment-naive males exhibited significantly higher endothelial Ki-67 expression compared to females (*p* = 0.031; BH-adjusted *p* = 0.103; Figure 3f), whereas no significant difference between sexes was found in the ADA-treated group (Figure 6).

For endothelial apoptosis, no differences were found between sexes in the treatment-naive group, while males had significantly higher endothelial cleaved Caspase-3 expression compared to females in the ADA-treated group (*p* = 0.039; BH-adjusted *p* = 0.078; Figure 4f, Figure 6).

Concerning vimentin expression, treatment-naive males had significantly higher vimentin area percentage compared to treatment-naive females (*p* = 0.017; BH-adjusted *p* = 0.085; Figure 5f), while no significant difference between sexes was found in the ADA-treated group (Figure 6). It is important to note that ADA treatment was associated with a significant increase in vimentin expression in females (*p* = 0.008; BH-adjusted *p* = 0.080) but not in males (*p* = 0.196).

These exploratory data collectively suggest that males may have more pronounced vascular changes in HS lesions than females at baseline (treatment-naive group), with higher endothelial proliferation and vimentin expression. Additionally, ADA treatment appears to be associated with more pronounced vascular differences in males, including lower vascular density compared to females and higher endothelial cell apoptosis following TNF-α blockade.

## 4. Discussion

Our study provides evidence that TNF-α signaling inhibition with ADA modifies the vascular microenvironment in HS lesions. The reductions in endothelial marker expression (CD31, vWF) and vascular density in ADA-treated lesions are consistent with suppression of angiogenic activation. In parallel, we observed increased vimentin expression and CD31-positive cells outside vascular spaces in the ADA-treated lesions, the significance of which remains to be further elucidated. Our exploratory findings also indicate that vascular changes in HS lesions after TNF-α blockade are sex-dependent, with male patients exhibiting a more pronounced baseline angiogenic profile and a greater decrease in vascular density than female patients. These results support the concept of HS as a long-term, immune-mediated disorder characterised by dysregulated cytokine signaling that sustains inflammation, tissue destruction, and fibrosis [1,2,3,4,5,6].

Endothelial activation and abnormal angiogenesis are increasingly recognized as significant contributors to cutaneous inflammation. In HS, the microvasculature is exposed to a milieu rich in TNF-α, IL-17, IL-1β, IL-8, and complement fragments, which enhance endothelial permeability, leukocyte recruitment, and disorganized vascular proliferation [6,11,31]. Our observation that vessel density is significantly reduced in ADA-treated lesions is consistent with TNF-α blockade attenuating this inflammatory angiogenic pathway. To investigate the mechanisms underlying this reduction in vascular density, we assessed endothelial cell proliferation (Ki-67) and apoptosis (cleaved Caspase-3) specifically within blood vessels. No significant differences were found in the percentage of Ki-67-positive or cleaved Caspase-3-positive endothelial cells between ADA-treated and treatment-naive groups, indicating that the reduction in vascular density is not mediated by direct suppression of endothelial proliferation or induction of endothelial apoptosis. This finding has important implications: it suggests that ADA reduces vascular density indirectly, most likely by attenuating the pro-angiogenic inflammatory milieu (TNF-α, VEGF-A, IL-8, IL-17) that drives new vessel formation, rather than by directly damaging or killing existing endothelial cells [6,11,31]. However, it is important to note that tissue and plasma levels of these pro-angiogenic mediators (VEGF-A, IL-17, IL-8, Ang-2, Tie2-axis components) were not measured in this study; the proposed mechanistic link between TNF-α blockade and reduced angiogenic drive therefore remains an inference supported by the existing literature rather than a directly demonstrated mechanism in our dataset. Notably, our cross-sectional design captures a snapshot of vessels that have already undergone remodeling; the endothelial cells present in ADA-treated lesions represent the surviving, functional vasculature, and their preserved proliferative and apoptotic indices suggest that these remaining vessels are viable. This is consistent with the concept of vascular remodelling, in which anti-inflammatory therapy may replace chaotic, immature inflammatory vessels with fewer, more stable ones [11,32].

In psoriasis, Hanssen et al. [11] demonstrated a reduction in endothelial Ki-67 expression following ADA treatment using a longitudinal paired design with pre- and post-treatment biopsies from the same patients. Our cross-sectional comparison across patients would not be expected to capture this dynamic change, as we are comparing the endpoint state of two groups rather than tracking changes within individuals over time. Moreover, the vascular pathology in HS is structurally distinct from that in psoriasis: HS involves deeper dermal and subcutaneous vasculature, tunnel formation, and peri-follicular fibrosis, which may influence the pattern and magnitude of vascular remodelling under TNF-α blockade compared to the more superficial dermal vasculopathy characteristic of psoriasis. Future longitudinal studies with paired biopsies in HS patients would be needed to determine whether ADA treatment induces a transient reduction in endothelial proliferation during the active phase of vascular remodelling.

Recent studies demonstrate that prolonged exposure to TNF-α paradoxically destabilizes the microvasculature, initially inducing angiogenesis but ultimately promoting endothelial apoptosis, barrier disintegration, and pro-thrombotic activation [33]. In ex vivo models, TNF-α stimulates the production of angiopoietin-2 and disrupts Tie2 signaling, leading to increased vascular permeability and the disruption of vascular quiescence [33]. Clinical research on psoriasis has demonstrated that TNF-α suppression reduces vascular diameter, cell proliferation, and irregular blood flow, suggesting that this effect is consistent across inflammatory skin disorders [11,34]. Our results are consistent with these concepts and suggest that ADA treatment in HS is associated with vascular changes toward a phenotype more characteristic of non-inflamed skin. These vascular improvements may impact clinical outcomes, particularly since HS lesions display edema, prolonged drainage, and painful swelling—symptoms indicative of vascular permeability and inflammatory congestion [1,35]. Notably, the ADA-treated group had significantly more severe disease at baseline, as reflected by higher Hurley staging (45.5% Hurley III vs. 26.3%; *p* = 0.007), higher IHS4 scores (*p* = 0.021), and longer disease duration (*p* = 0.002). Since more severe and chronic HS is associated with greater inflammatory burden, increased angiogenesis, and more extensive tissue remodeling, one would expect higher—not lower—vascular density in the ADA-treated group if treatment had no effect. The finding that ADA-treated lesions nonetheless exhibited significantly lower vascular density despite more severe baseline disease strengthens the interpretation that TNF-α blockade is associated with reduced angiogenic activity. However, without paired pre- and post-treatment biopsies, this interpretation remains associative rather than causal. The prevalence of smoking was notably high in both groups (73.0% vs. 81.8%; *p* = 0.409), consistent with the well-established association between smoking and HS. While smoking is known to promote endothelial activation and angiogenesis through VEGF upregulation, this factor did not differ between groups and is therefore unlikely to account for the observed between-group differences in vascular density. Similarly, BMI did not differ significantly between groups (26.8 ± 5.3 vs. 28.4 ± 5.7; *p* = 0.312), although approximately one-third of patients were obese (BMI ≥ 30), a condition associated with chronic low-grade inflammation and endothelial dysfunction. Data on diabetes mellitus, hypertension, and dyslipidemia were not available and represent unmeasured confounders that may influence vascular biomarker expression.

An interesting exploratory finding was the elevated baseline vascular density and vimentin levels in male compared to female treatment-naive patients. Although HS is more prevalent in women, male patients frequently have more severe, fibrotic, and deeply tunnelling disease [1,36]. Our findings align with clinical observations and recent molecular studies that show sex-dependent variation in immune and vascular biology [37]. Hormonal influences may contribute to these sex differences as estrogen improves the integrity of blood vessels and regulates fibroblast growth, while androgens tend to increase fibroblast numbers and ECM production [38,39,40,41]. However, hormonal levels (estrogen and androgens) and other potential sex-related confounders were not measured in this study; the sex-dependent findings therefore represent associations for which causality cannot be established. These results highlight the significance of incorporating sex as a biological variable in HS pathogenesis and therapeutic response. The Ki-67 and cleaved Caspase-3 data add a mechanistic dimension to these sex-related differences. Treatment-naive males exhibited significantly higher endothelial Ki-67 expression compared to females, while no sex difference was found in the ADA-treated group. This suggests that male HS lesions have a more proliferatively active endothelium at baseline, consistent with the higher vascular density observed in treatment-naive males. The loss of this sex difference in the ADA-treated group suggests that ADA may normalize the hyperproliferative endothelial phenotype seen in males, bringing their proliferation levels closer to those of females.

Conversely, endothelial apoptosis showed the opposite pattern: no sex difference was found in treatment-naive lesions, but in the ADA-treated group, males had significantly higher cleaved Caspase-3 expression than females. This emergence of a sex difference after treatment is striking and suggests that ADA may differentially affect endothelial cell survival in a sex-dependent manner. In males, TNF-α blockade appears to unmask or induce endothelial apoptosis, which, combined with the significantly lower vascular density in ADA-treated males compared to females, may partly explain why males show a more pronounced vascular density reduction following treatment. In females, the notably low cleaved Caspase-3 expression in ADA-treated lesions suggests that female endothelial cells may achieve a more quiescent, stabilized state following TNF-α blockade. These findings are consistent with known sex-related differences in vascular biology: estrogen promotes endothelial stability, reduces vascular permeability, and enhances endothelial survival signaling, whereas androgens promote endothelial proliferation and angiogenesis but may also increase vascular susceptibility to apoptotic stimuli during inflammatory resolution [38,40,41].

Regarding vimentin expression, treatment-naive males had significantly higher levels than females, while this difference disappeared in the ADA-treated group, driven by a significant increase in vimentin expression in females but not males. The biological significance of this sex-dependent change remains unclear. We hypothesize that the increased vimentin signal in ADA-treated females may reflect enhanced fibroblast activation, stromal remodeling, or possibly endothelial–mesenchymal transition, but distinguishing among these possibilities would require co-staining with fibroblast-specific markers (e.g., platelet-derived growth factor receptor (PDGFRα), fibroblast activation protein(FAP)) and EndMT markers (e.g., SNAI1, N-cadherin), combined with compartment-specific automated quantification. This remains an important direction for future investigation.

Collectively, the sex-stratified data reveal a pattern in which sex-dependent differences in vascular markers either disappear or newly emerge following ADA treatment, suggesting that the sexes respond to TNF-α blockade through partially distinct mechanisms. Males appear to undergo more pronounced vascular regression (lower vessel density, higher apoptosis), while females show a greater increase in vimentin expression. This pattern—in which significant sex differences in the treatment-naive group vanish after treatment (Ki-67, vimentin) and non-significant differences become significant (apoptosis, vascular density)—underscores the importance of considering sex as a biological variable in HS research and may have implications for personalized treatment strategies. Stratified therapy approaches may ultimately improve clinical outcomes [9,31,42].

Several important limitations of our study must be acknowledged. First, the cross-sectional design and lack of paired pre- and post-treatment biopsies preclude causal inference; between-patient heterogeneity in disease duration, severity, anatomic site, prior therapies, and comorbidities may contribute to the observed differences in vascular markers. Second, the clinical characterization of our cohort is limited: standardized disease severity scoring (Hurley staging, IHS4, HiSCR status) was available and revealed significantly higher Hurley staging and IHS4 scores in the ADA-treated group compared to the treatment-naive group, which is expected given that adalimumab is indicated for moderate-to-severe disease. While this severity imbalance could confound between-group comparisons, it would be expected to bias against finding lower vascular density in ADA-treated lesions, as more severe disease is associated with greater angiogenic activity. Data on lesion chronicity, smoking status, BMI, and metabolic syndrome were collected (Table 2) and showed no significant between-group differences, but other potential confounders, including diabetes mellitus, hypertension, and dyslipidemia, were not collected and represent unmeasured variables that could influence vascular biomarker expression. Although BMI and smoking status did not differ between groups, the high overall prevalence of smoking (77.1%) and obesity (32.9%) in our cohort may independently affect vascular biology and should be considered when interpreting these findings. Third, pharmacokinetic data (ADA trough levels, anti-drug antibodies, precise time since last dose) were not collected, preventing exposure–response analysis and the exclusion of under-exposure or reverse causality as confounding factors. Fourth, the vimentin quantification was performed across whole visual fields and does not differentiate between endothelial and stromal compartments; compartment-specific analysis (e.g., using cell-type masks or spatial transcriptomics) would be needed to definitively attribute vimentin changes to specific cell types. Fifth, the assessment of Ki-67 and cleaved Caspase-3 was based on manual classification of endothelial cells within a limited number of vessel profiles per sample (range: 5–12), which may limit statistical power and inflate variance. The relatively high Ki-67 fractions observed (28–33%) are consistent with the hyperproliferative state of actively inflamed HS microvasculature, but we cannot entirely exclude the possibility that occasional non-endothelial nuclei (e.g., closely apposed pericytes or inflammatory cells) were included despite our CD31 colocalization criterion. Sixth, tissue and plasma levels of pro-angiogenic mediators (VEGF-A, IL-17, IL-8, Ang-2, Tie2-axis components) and functional vascular assessments (permeability, perfusion) were not performed, limiting our ability to directly demonstrate the proposed indirect anti-angiogenic mechanism. Seventh, the sex-stratified analyses were exploratory and involved multiple comparisons without formal correction; the modest subgroup sizes (*n* = 15–20 per subgroup) limit statistical power, and the sex-dependent signals (*p* = 0.031–0.039) are potentially susceptible to type I error at this testing density. Cleaved Caspase-3 specifically detects the execution phase of apoptosis; earlier apoptotic events or alternative cell death pathways (e.g., ferroptosis, necroptosis) were not assessed. Future studies should integrate longitudinal sampling with paired biopsies, single-cell and spatial transcriptomics, automated digital vascular phenotyping, therapeutic drug monitoring, quantitative collagen morphometry (building on the qualitative Picrosirius Red and Mallory’s Trichrome findings presented in Supplementary Figures S1 and S2), and comprehensive clinical phenotyping to enhance the understanding of how TNF-α inhibition restructures the HS microenvironment [6,43]. Combination therapies targeting TNF-α together with IL-1, IL-17, or anti-fibrotic pathways may provide greater therapeutic benefit [31,44].

## 5. Conclusions

This study provides tissue-level evidence that ADA-treated HS lesions are associated with reduced angiogenic markers, indicating a disease-modifying effect that extends beyond inflammatory cytokine suppression and reduction of inflammatory cell numbers. Importantly, the reduction in vascular density was not shown to be mediated by direct suppression of endothelial cell proliferation or induction of apoptosis, as Ki-67 and cleaved Caspase-3 expression in endothelial cells did not differ significantly between treatment groups. This is consistent with ADA acting indirectly on the vasculature, most likely by attenuating the pro-angiogenic inflammatory milieu, although this proposed mechanism requires direct confirmation through measurement of tissue and plasma angiogenic mediators in future studies. Sex-stratified analysis revealed that treatment-naive males have a more proliferatively active and vascularized lesional microenvironment, and that ADA treatment differentially affects endothelial turnover in a sex-dependent manner, with males showing higher post-treatment apoptosis. The observed increase in vimentin expression in ADA-treated lesions warrants further investigation to determine its biological significance. Our results underscore the therapeutic potential of targeting vascular dysregulation in HS and indicate that sex-related biological differences may affect vascular responses and treatment outcomes. Future research utilizing longitudinal sampling, molecular profiling, and integrated pathway targeting may enhance the understanding of vascular remodeling mechanisms and promote the development of individualized therapeutic approaches.

## Figures and Tables

**Table 1:** Primary and secondary antibodies used for immunofluorescence staining.

Antibody		Catalog Number	Host	Dilution	Source
Primary	CD31 (PECAM-1) (89C2)	#3528S	Mouse	1:400	Cell Signaling Technology (CST), (Danvers, MA, USA)
	Vimentin (D21H3) XP^®^ mAb	#5741S	Rabbit	1:300	Cell Signaling Technology (CST), (Danvers, MA, USA)
	vWF (D8L8G) XP^®^ mAb	#65707S	Rabbit	1:400	Cell Signaling Technology (CST), (Danvers, MA, USA)
	Anti-αSMA	M0851	Mouse	1:500	DAKO, Glostrup, Denmark
	Anti-Ki-67 Antibody	AB9260	Rabbit	1:100	Chemicon International (Temecula, CA, USA)
	Cleaved Caspase 3 Polyclonal antibody	25128-1-AP	Rabbit	1:500	Proteintech Group (Rosemont, IL, USA)
Secondary	Alexa Fluor^®^ 488 AffiniPure^®^ Donkey Anti-Rabbit IgG (H + L)	711-545-152	Donkey	1:400	Jackson Immuno Research Laboratories, Inc., (West Groove, PA, USA)
	Rhodamine Red™-X (RRX) AffiniPure™ Donkey Anti-Mouse IgG (H + L)	715-295-151	Donkey	1:400	Jackson Immuno Research Laboratories, Inc., (West Groove, PA, USA)

**Table 2:** Clinical characteristics of the patient cohort.

Parameter	Treatment-Naive (*N* = 38)	ADA-Treated (*N* = 33)	*p*-Value
Sex, *N* (%)			1.000 *
Male	20 (52.63)	18 (54.55)	
Female	18 (47.37)	15 (45.45)	
Age, years (mean ± SD)	34.7 ± 10.9	39.2 ± 11.7	0.100 ^†^
BMI (mean ± SD)	26.8 ± 5.3	28.4 ± 5.7	0.312 ^‡^
Smoking, *N* (%)			0.409 *
Yes	27 (73.0)	27 (81.8)	
No	10 (27.0)	6 (18.2)	
Hurley stage, *N* (%)			0.007 *
I	14 (36.8)	2 (6.1)	
II	14 (36.8)	16 (48.5)	
III	10 (26.3)	15 (45.5)	
IHS4 score (mean ± SD)	8.5 ± 7.4	12.2 ± 7.2	**0.021 ^‡^**
Disease duration, years (mean ± SD)	6.6 ± 7.0	12.2 ± 10.0	**0.002 ^‡^**
Biopsy region, N (%)			0.510 *
Axilla	17 (44.74)	17 (51.52)	
Other	21 (55.26)	16 (48.48)	

* Chi-square test with Fisher’s exact correction; ^†^ unpaired *t*-test with Welch’s correction; ^‡^ Mann–Whitney U test. Bold *p*-values indicate statistical significance (*p* < 0.05).

## Data Availability

The data presented in this study are available from the corresponding author upon reasonable request.

## Supplementary Materials

The following supporting information can be downloaded at: https://www.mdpi.com/article/10.3390/biomedicines14040741/s1, Figure S1: Picrosirius Red staining of control, treatment-naive, and adalimumab-treated hidradenitis suppurativa skin; Figure S2: Mallory’s Trichrome staining of control, treatment-naive, and adalimumab-treated hidradenitis suppurativa skin.

## Abbreviations

The following abbreviations are used in this manuscript:

ADA	adalimumab
αSMA	alpha-smooth muscle actin
BMI	Body Mass Index
CD31	cluster of differentiation 31
CST	cell signaling technology
CTRL	control tissue
DAPI	4′,6-diamidino-2-phenylindole
ECM	extracellular matrix
EndMT	endothelial–mesenchymal transition
FAP	fibroblast activation protein
FFPE	formalin-fixed, paraffin-embedded
HiSCR	Hidradenitis Suppurativa Clinical Response
H&E	hematoxylin and eosin
HS	hidradenitis suppurativa
IHC	immunohistochemistry
IHS4	International Hidradenitis Suppurativa Severity Score System 4
IL	interleukin
IF	immunofluorescence
NIH	National Institutes of Health
PBS	phosphate-buffered saline
PDGFRα	platelet-derived growth factor receptor alpha
PECAM-1	platelet endothelial cell adhesion molecule 1
PFA	paraformaldehyde
ROI	region of interest
SD	standard deviation
SNAI1	snail family transcriptional repressor 1
Tie2	tyrosine kinase with immunoglobulin and EGF homology domains 2
TNF-α	tumor necrosis factor alpha
VEGF-A	vascular endothelial growth factor
vSMC	vascular smooth muscle cell
vWF	von Willebrand factor
WSI	whole-slide images
