# Supplementary material for: Adalimumab Treatment Modulates Vascular Changes in Hidradenitis Suppurativa Lesions in a Sex-Dependent Manner
Source: Biomedicines. 2026 Mar 24;14(4):741. doi: 10.3390/biomedicines14040741 (PMC13114255; doi:10.3390/biomedicines14040741)
Supplement: Supplementary file 1 [file biomedicines-14-00741-s001.zip › Supplementary materials/Supplementary material.pdf]

biomedicines

MDPI

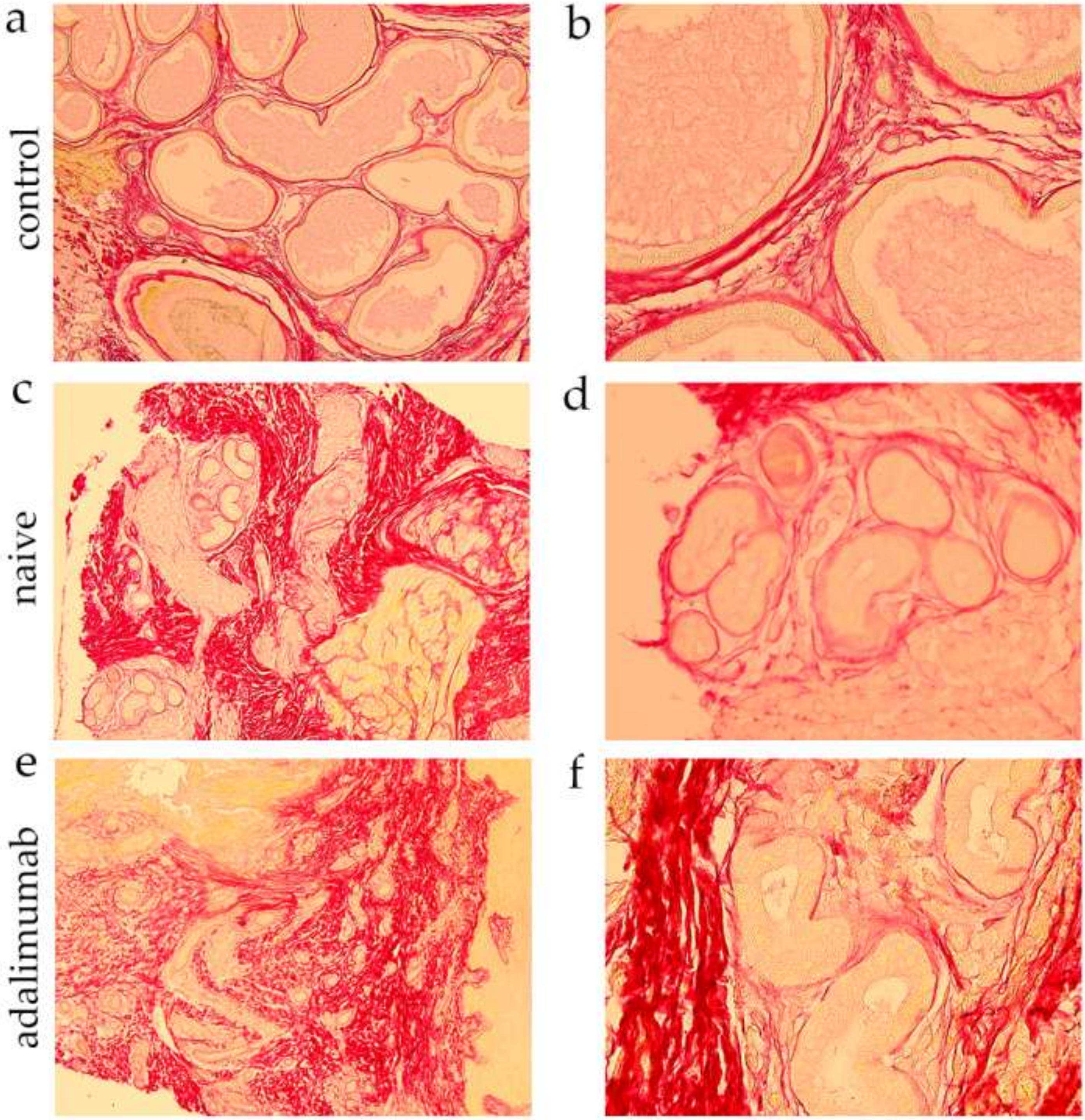

Supplementary Figure S1. Picrosirius Red staining of control, treatment-naive, and adalimumab-treated hidradenitis suppurativa skin. (a, b) Control skin displays organized collagen fibers (red) surrounding sebaceous and sweat glands, with well-preserved dermal architecture. (c, d) Treatment-naive HS lesions show markedly increased collagen deposition (dense red staining) with fibrotic compression of adnexal structures, consistent with chronic inflammatory fibrosis. (e, f) ADA-treated HS skin exhibits collagen staining with better-preserved glandular architecture compared to treatment-naive lesions. Left panels (a, c, e) show lower magnification overview (×10);

right panels (b, d, f) show higher magnification of dermal structures (×40). Collagen fibers stain red; background tissue appears pale yellow.

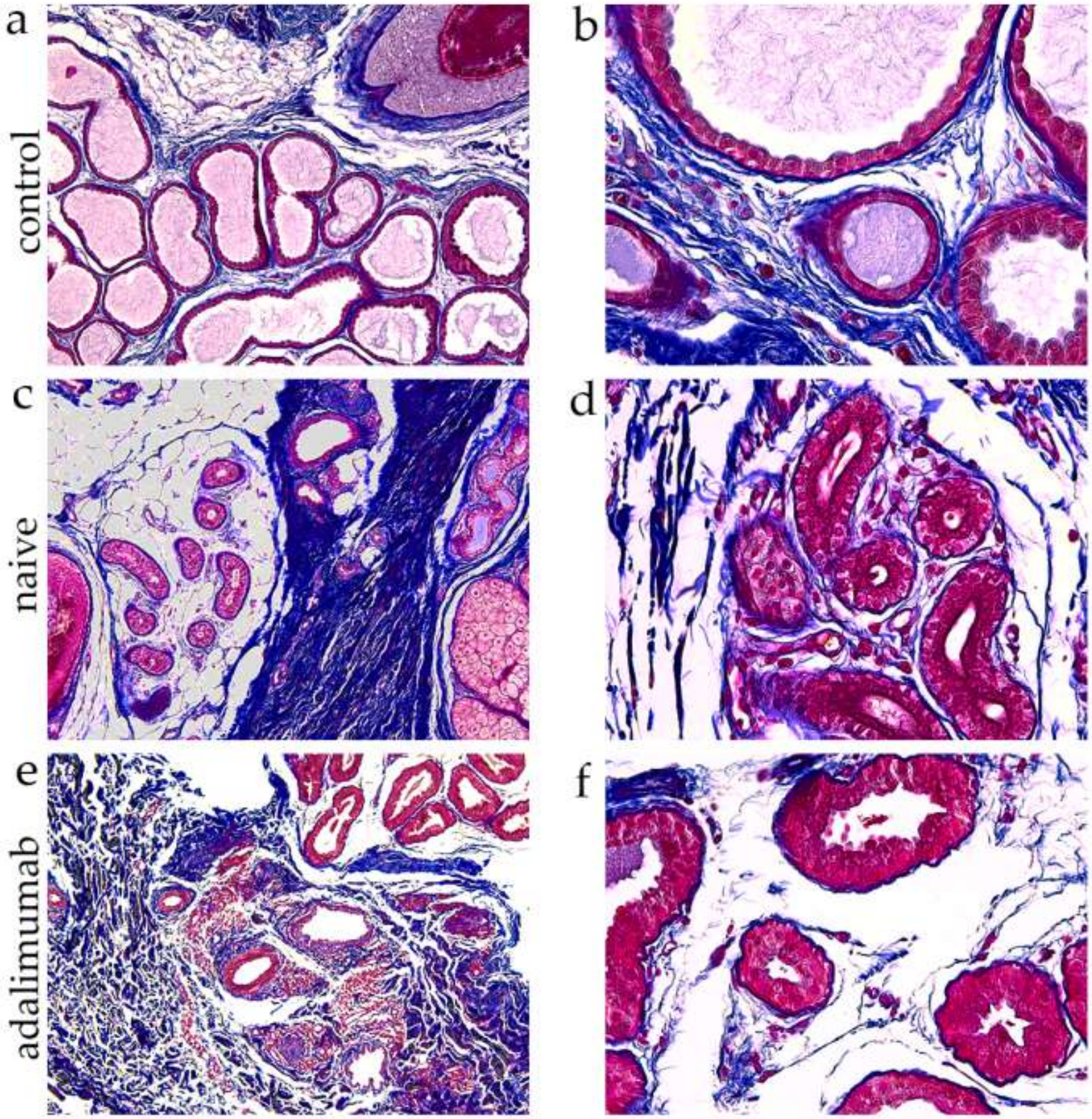

Supplementary Figure S2. Mallory's Trichrome staining of control, treatment-naive, and adalimumab-treated hidradenitis suppurativa skin. (a, b) Control skin displays organized collagen fibers (blue) surrounding sebaceous and sweat glands, with well-preserved dermal architecture. (c, d) Treatment-naive HS lesions show markedly increased collagen deposition (dense blue staining) with fibrotic compression of adnexal structures, consistent with chronic inflammatory fibrosis. (e, f) ADA-treated HS skin exhibits an intermediate pattern of collagen deposition, with less dense fibrosis compared to treatment-naive lesions and better-preserved glandular architecture. Left panels (a, c, e) show lower magnification overview (×10); right panels (b, d, f) show higher magnification of

dermal structures (×40). Collagen fibers stain blue, muscle fibers and cytoplasm stain red, and nuclei stain dark brown/black.

https://doi.org/10.3390/xxxxx
