# Supplementary figures and images for: Adalimumab Treatment Modulates Vascular Changes in Hidradenitis Suppurativa Lesions in a Sex-Dependent Manner
Source: Biomedicines. 2026 Mar 24;14(4):741. doi: 10.3390/biomedicines14040741 (PMC13114255; doi:10.3390/biomedicines14040741)

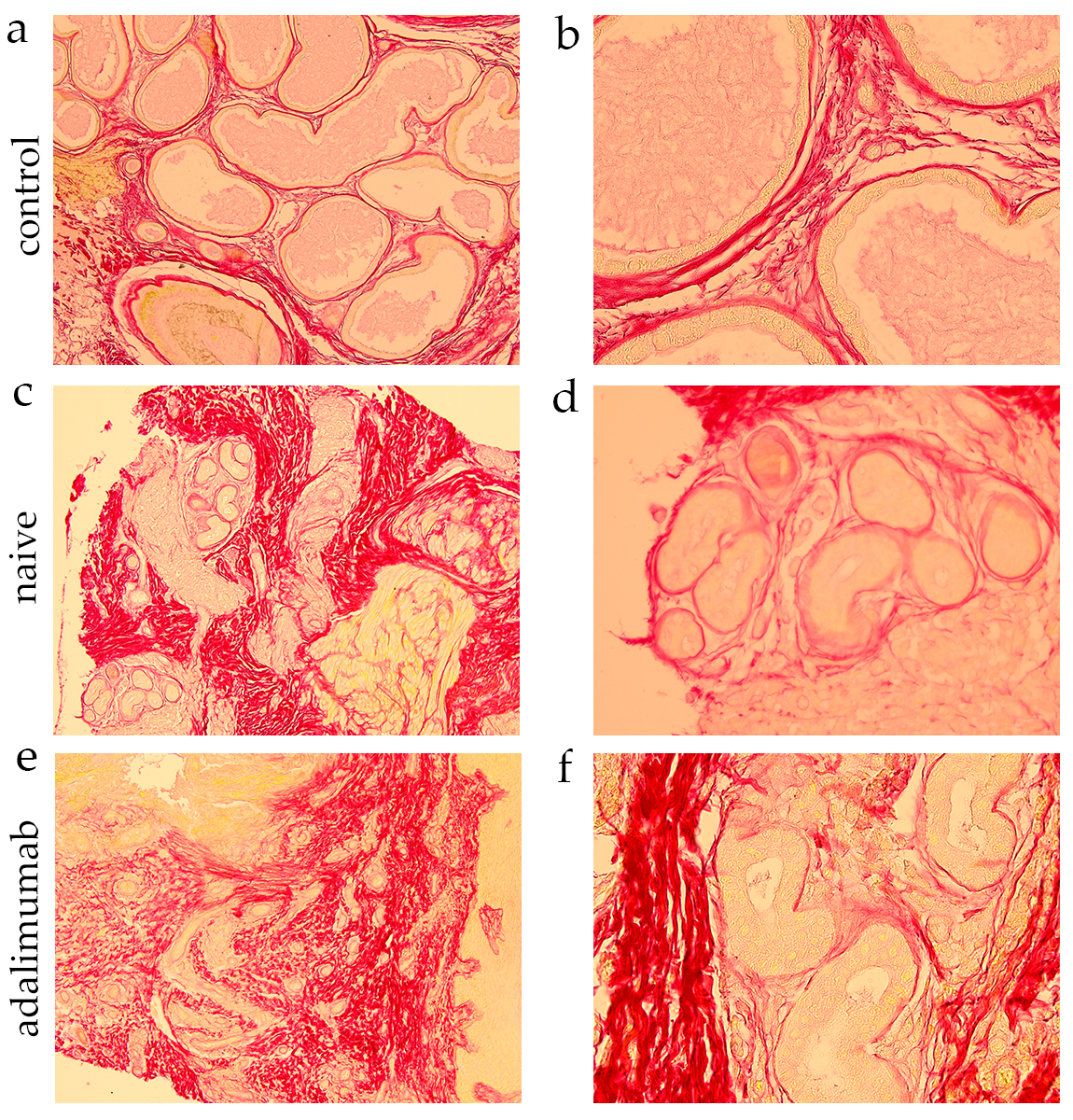

Supplement: Supplementary file 1 [file biomedicines-14-00741-s001.zip › Supplementary materials/Figure S1.tif]

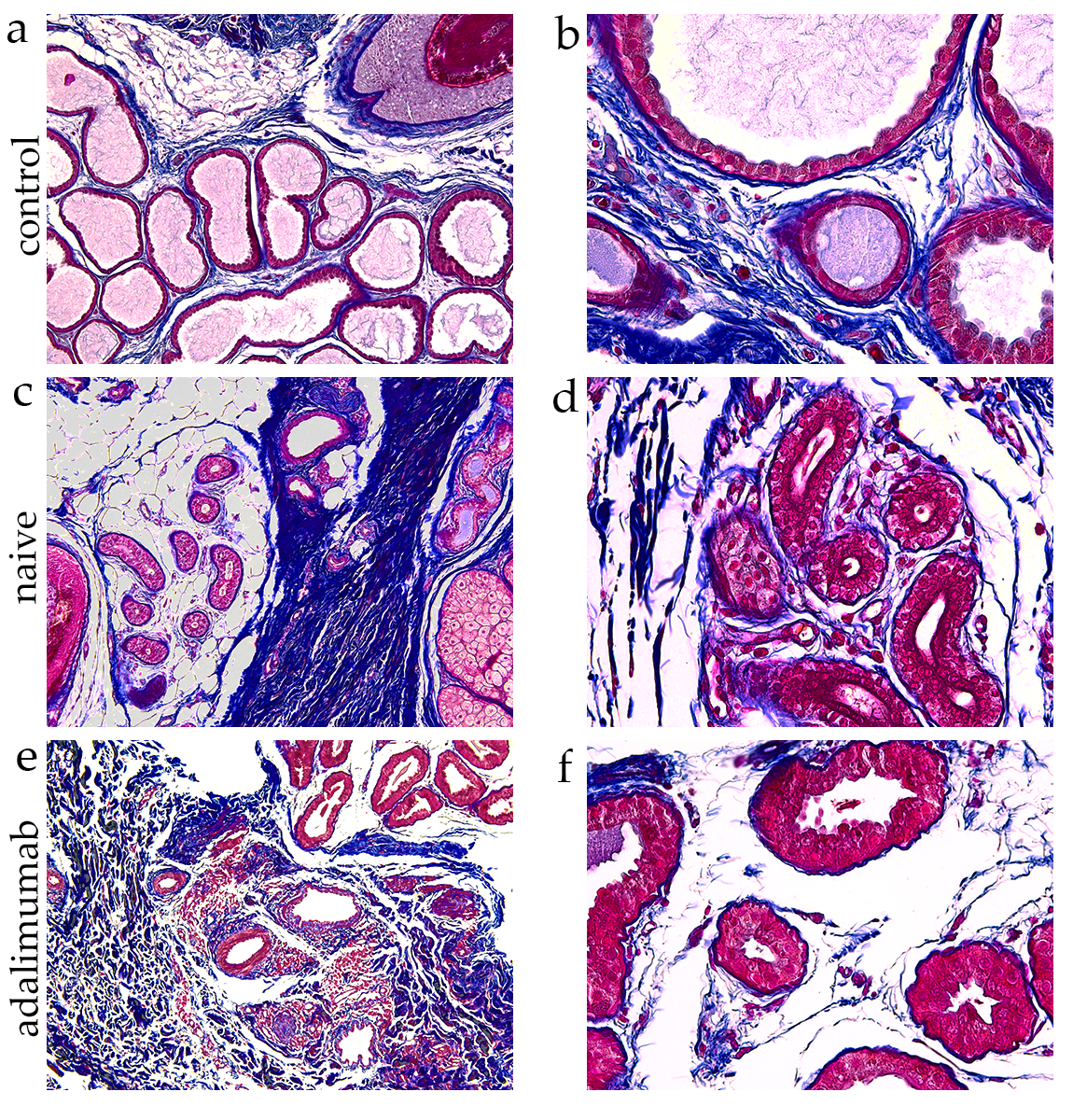

Supplement: Supplementary file 1 [file biomedicines-14-00741-s001.zip › Supplementary materials/Figure S2.tif]
